# The relationship between loneliness and depression among college students: Mining data derived from passive sensing

**DOI:** 10.1177/20552076231211104

**Published:** 2023-11-06

**Authors:** Malik Muhammad Qirtas, Evi Zafeiridi, Eleanor Bantry White, Dirk Pesch

**Affiliations:** 1 School of Computer Science and Information Technology, University College Cork, Cork, Ireland; 2 School of Applied Social Studies, University College Cork, Cork, Ireland; 3 School of Computer Science and Information Technology, University College Cork, Cork, Ireland

**Keywords:** Loneliness, depression, passive sensing, smartphone, students, fitness tracker

## Abstract

**Background:**

While there is recognition of the relationship between loneliness and depression, specific behavioural patterns distinguishing both are still not well understood.

**Objectives:**

Our objective is to identify distinct behavioural patterns of loneliness and depression from a passively collected dataset of college students, understand their similarities and interrelationships and assess their effectiveness in identifying loneliness and depression.

**Methods:**

Utilizing the StudentLife dataset, we applied regression analysis to determine associations with self-reported loneliness and depression. Mediation analysis interprets the relationship between the two conditions, and machine learning models predict loneliness and depression based on behavioural indicators.

**Results:**

Distinct behavioural patterns emerged: high evening screen time (OR = 1.45, p = 0.002) and high overall phone usage (OR = 1.50, p = 0.003) were associated with more loneliness, whereas depression was significantly associated with fewer screen unlocks (OR = 0.75, p = 0.044) and visits to fewer unique places (OR = 0.85, p = 0.023). Longer durations of physical activity (OR = 0.72, p = 0.014) and sleep (OR = 0.46, p = 0.002) are linked to a lower risk of both loneliness and depression. Mediation analysis revealed that loneliness significantly increases the likelihood of depression by 48%. The prediction accuracy of our XGBoost-based machine learning approach was 82.43% for loneliness and 79.43% for depression.

**Conclusion:**

Our findings show that high evening screen time and overall phone usage are significantly associated with increased loneliness, while fewer screen unlocks and visits to fewer unique places are significantly related to depression. The findings can help in developing targeted interventions to promote well-being and mental health in students.

## Introduction

Loneliness and depression are important public health concerns that affect people's mental and physical health. Loneliness is increasing globally, with many identifying it as their principal cause of unhappiness.^1^ Loneliness is an experience in which a person perceives a lack of quality social relationships.^2^ Both the World Health Organization and the US Surgeon General have highlighted the ‘epidemic’ of loneliness, emphasizing its role as a substantial risk factor for numerous health conditions.^3,4^ These institutions have prioritized understanding and addressing loneliness as a key concern in public health. Depression, or major depressive disorder (MDD), is a prominent health problem, characterized by persistent feelings of sadness, despair, and lack of interest in engaging in activities.^5^ Depression may lead to suicide, which takes over 800,000 lives every year, the equivalent to one life every 40 seconds throughout the globe.^6^ The COVID-19 pandemic has had a significant impact on people's mental health. Widespread lockdowns and social isolation have made people feel more lonely than usual.^7^ Although they each have their own unique characteristics, loneliness and depression are interrelated conditions that can influence and exacerbate each other.

The relationship between them is well established, but the understanding of the specific behavioural patterns that distinguish loneliness and depression is still limited.^8,9^ Given the substantial and significant effects of loneliness and depression on both personal health and broader societal functioning, it is of vital importance to enhance our understanding of their interrelationship. This will enable us to unravel the complex dynamics at play and devise effective strategies for intervention and support. However, this study narrows its focus to investigating specific behavioural traits, identified through passively sensed data from smartphones, and their distinct or shared impact on loneliness and depression. No previous study has used passive smartphone data to explore the relationship between these two mental health conditions. Our work aims to fill this gap, offering new insights into how behavioural patterns, detected by smartphone sensors, can reveal the relationship between feelings of loneliness and depression.

Passive sensing, utilizing smartphone and wearable data, opens new avenues for detecting loneliness and depression. This method offers benefits such as being unobtrusive, real-time, and cost-effective. Digital biomarkers, derived from passive sensing data along with validated established psychometric scales, allow the creation of prediction models to identify individuals experiencing the loneliness or depression.^10,11^ Unlike traditional methods that focus on individual conditions, passive sensing provides continuous, longitudinal data to understand how loneliness and depression interrelate and change over time. This approach has the potential to enhance early detection and intervention, leading to more effective and targeted support for those at risk.

College students are at a higher risk of experiencing loneliness and depression, two interconnected mental health issues.^8^ It is essential to understand the particular circumstances in which college students experience loneliness and depression. These issues may be exacerbated by college life, which is defined by new experiences, academic pressures and the need for social integration.^12^ The combination of higher stress levels and the transition to adulthood makes college students more prone to mental health issues. The impact of loneliness and depression on academic progress and well-being of students is significant.^13^ By understanding the distinct effects of loneliness and depression on college students, we can tailor interventions to meet their specific needs and challenges.

While some studies have investigated the link between loneliness and depression, what distinguishes our study is its focus on providing granular details of behaviours associated with each condition using passive sensing data, specifically in college students, something that has not been extensively explored in the existing literature. Studies have suggested a bidirectional relationship between these mental health issues,^14^ but the dynamics in a college context require further examination. Similarly, while Qualter et al. established that loneliness predicted depressive symptoms among adolescents,^15^ the relationship's intricacy among college students remains underexplored in terms of identifying specific behavioural patterns associated with each condition using passive sensing. Additionally, Stickley et al. demonstrated an association between loneliness and depression in a cross-sectional study,^16^ but longitudinal research using passive sensing data could provide valuable insights into their complex relationship. These findings emphasize the necessity for more research on loneliness and depression among college students, particularly by leveraging passive sensing data to address this research gap.

Utilizing regression analysis, mediation analysis, association rule mining (ARM), and machine learning analysis, we explored the relationship between loneliness and depression and student behaviours, focusing specifically on predictive digital biomarkers such as screen time, phone usage, physical activity durations, and sleep patterns. We applied these approaches to a specific dataset known as the StudentLife dataset. The research questions explored in our study were the following:

1. What behavioural patterns are common in students who are lonely or depressed, and how do these differ from those who are not?
2. How do loneliness and depression directly and indirectly affect each other in the context of college students, and which specific behavioural features serve as mediators in these relationships?
3. How effectively can behavioural features, when combined with loneliness and depression scales as an input feature, identify students with feelings of loneliness or symptoms of depression?

## Methods

### Dataset

We used a subset of the Student Life dataset, which is data passively collected using smartphone sensors from students at Dartmouth College.^17^ The dataset used can be found at https://studentlife.cs.dartmouth.edu/dataset.html. All participants were recruited at Dartmouth College in the Spring of 2013. The study was approved by the Institutional Review Board at Dartmouth College. The study was authorized by Dartmouth College's Institutional Review Board. Thirty undergraduates and 18 graduate students comprise the 48 students who completed the study. The data gathering phase lasted 10 weeks during the whole spring semester. Automatic sensor data is gathered and transferred to the cloud while the phone is being recharged and connected to WiFi. During the data collection phase, students were asked to answer several Ecological Momentary Assessment (EMA)-related questions while using their mobile devices. The data includes activity data (activity duration, indoor/outdoor mobility), conversation data (duration and frequency), sleep data (duration, onset, and waking time), and location data (GPS, indoor building, and co-located Bluetooth devices).

### Loneliness and depression scales

The StudentLife dataset also includes measures of loneliness and depression, assessed using the UCLA Loneliness Scale and the Patient Health Questionnaire-9 (PHQ-9), respectively. The UCLA Loneliness Scale is a 20-item questionnaire designed to evaluate feelings of social isolation, emptiness, and dissatisfaction with one's current social relationships.^18^ This scale features 10 positive items and 10 negative items, with a scoring range of 20–100. Higher scores signify higher levels of loneliness, and scores exceeding 43 suggest a strong sense of loneliness. In the sample data, scores ranged from a low of 25 to a high of 64.

In terms of depression assessment, the dataset contains pre- and post-surveys based on the PHQ-9 scale. The PHQ-9 is a widely used, self-administered questionnaire that measures the severity of depressive symptoms. Comprising nine items, it examines the frequency and intensity of symptoms such as loss of interest or pleasure, changes in eating or sleep patterns, feelings of worthlessness or guilt, difficulties concentrating, fatigue, and thoughts related to suicide or death. Each item is scored on a scale of 0 (never) to 3 (almost daily), resulting in a total score ranging from 0 to 27. A score above 9 indicates high depression, while a score between 0 and 9 signifies low depression. This simplification is based on the finding that a PHQ-9 cutoff of 10 has more discriminating power to diagnose depression, which allows for a more straightforward comparison between groups of students experiencing low or high levels of depressive symptoms.^19^ It is important to note that the PHQ-9 scale can be used to identify a more nuanced range of depression severity levels, but the binary distinction was considered appropriate in this context to focus on the key differences in behavioural features between the two groups as used by other studies.^20,21^ The PHQ-9 has been validated for various contexts and populations and is extensively employed in clinical and research settings for depression screening, monitoring symptom severity over time and evaluating the effectiveness of treatments.^22^

### Data preprocessing

During the data preprocessing phase, we applied specific inclusion criteria for selecting our study sample. Our inclusion criteria consisted of students who completed both the post-study loneliness and depression questionnaires, and we excluded students who did not complete either or both loneliness and depression post-study questionnaires. This comprehensive approach allowed us to ensure that we had a robust and reliable measure of each participant's perceived loneliness and depression at the end of the study period, which served as the ground truth in our study. The use of this criteria led to a final sample of 41 students for our analysis.

The choice to focus on post-study measures, rather than pre-study surveys, was a conscious one. We believe that post-study measures better interpret and reflect the actual feelings of students regarding loneliness and depression throughout the semester. These feelings may be influenced or enhanced due to academic pressures such as exams or project deadlines. Therefore, we argue that the post-study measures offer a more accurate representation of the students’ mental state during the academic term.

We then converted the UNIX timestamps of each sensor's data into a human-readable local date and time format using each participant's timezone information. Recognizing that students engage in a variety of activities throughout the day, we divided a 24-hour period into three distinct sessions: day session (9 a.m.–6 p.m.), evening session (6 p.m.–12 a.m.) and night session (12 a.m.–9 a.m.). This allowed us to compute 24-hour day-level features along with the aforementioned epochs.

To handle missing values, we first removed all records containing outliers using the Z-score method.^23^ We then imputed missing continuous data for each participant using the median of the respective feature. For categorical data, we employed the mode of the corresponding feature. Since tree-based algorithms are not affected by feature scaling, we only scaled the numerical features for the other algorithms. We used the ‘standard scaler’ in our models, as it transforms the data to have a mean of 0 and a standard deviation of 1, effectively normalizing the data.

### Behavioural feature extraction

For the analysis in this study, we calculated a total of 82 features from the passively sensed smartphone dataset using the Reproducible Analysis Pipeline for Data Streams (RAPIDS) tool.^24^ RAPIDS is designed for data preprocessing and biomarker computation. These features can be classified into several categories based on the sensor data from which they were derived. The following sections provide a brief overview of each type of feature we extracted. Please refer to the RAPIDS for a detailed list and description of all the features used in this study.^24^

### Call log features

We processed and analyzed incoming/outgoing and missed calls from the passive sensing data. We focused on various aspects of call data, including call frequency, contact diversity, duration statistics, and the timing of calls. The key categories of features extracted are described below.

#### Call frequency and contact diversity

This includes the total count of calls, the number of distinct contacts, and the count of calls with the most frequent contact. These features were calculated for each epoch and day level. They provide insight into the social activity and network diversity of students.

#### Call duration

For each epoch and day level, we computed various statistics about the duration of calls, including the mean, sum, minimum, maximum, and standard deviation. We also calculated the mode and the entropy of call durations to capture the variability and predictability of call duration patterns.

#### Call timing

The timing of the first and last call was recorded for each epoch and day level, providing insight into the students’ daily rhythms and patterns of activity.

#### Missed calls

We also examined the frequency of missed calls, the diversity of contacts involved in missed calls, and the timing of these missed calls.

### SMS log features

For text message (SMS) data, we have focused on diverse aspects such as message frequency, contact diversity, and timing of messages. The key categories of these features are described as follows:

#### Message frequency and contact diversity

This category includes the total count of messages and the number of distinct contacts per each epoch and day level. Additionally, we computed the count of messages from the most frequent contact. These features serve as proxies for social engagement and the diversity of students’ social networks.

#### Message timing

The timing of the first and last messages was recorded for each epoch and day level, highlighting students’ communication patterns and active hours.

### GPS features

Our study also took advantage of the rich geospatial data available from students’ smartphones. We extracted two types of features: features related to home and general location and features focused on movements and significant and unique locations. These features provided a comprehensive view of students’ location habits and routines, as well as their movements and overall geographical behaviours.

#### Static location features

These features focused on the students’ behaviours at particular locations, such as their homes or frequently visited places. We derived the time spent at home and the number of frequent locations visited, among other features. Shannon's entropy, calculated based on the proportion of time spent at each frequent location, allowed us to assess the diversity of students’ location habits. These features shed light on how much time students spent in familiar environments versus exploring new ones, which could be linked to their mental well-being.

#### Dynamic movement features

This category included features that reflected the students’ movement patterns and speed. We calculated the total distance travelled in an epoch, average and variance of speed during movements and the radius of gyration, which provided a measure of the area covered by a student in a day. We also evaluated the ratio between stationary time and total location sensed time, which served as an indicator of how mobile or stationary a student's routine was.

### Phone usage features

Phone usage features offer valuable insights into students’ digital habits and behaviours, providing a unique lens to understand their daily life patterns.

#### Unlock episode features

These features provide insights into the general phone usage patterns by evaluating the frequency, duration, and timing of device unlocking episodes.

#### App usage features

These features focus on the usage of individual apps or app categories, providing detailed information about digital habits such as preferred applications, periods of high or low app activity and the diversity of apps used. This analysis not only accounts for the frequency of usage but also the duration and timing of app usage.

### Connectivity features: Bluetooth and WiFi

Extracting students’ connectivity habits encompassed data from both Bluetooth and WiFi sensors. Both provide unique insights into the environmental context and patterns of engagement with devices and networks.

#### Frequency of interactions

For both Bluetooth and WiFi, we computed the total number of scans in each epoch, capturing the number of unique devices or access points connected or sensed. These provide a measure of overall digital and social connectivity.

#### Diversity of interactions

Additionally, we considered the diversity of connections by counting the number of unique devices (in the case of Bluetooth) or access points (in the case of WiFi) accessed within a particular epoch. This measure helped elucidate the extent of their interactions and mobility.

#### Persistence of interactions

Finally, we looked at the persistence of certain connections by identifying the most frequently scanned device (for Bluetooth) or access point (for WiFi) in an epoch. This offered insights into dominant or preferred locations or devices.

### Sleep features

Sleep features are extracted to provide a detailed insight into the duration, quality and patterns of different sleep types. Here are the key features.

#### Sleep timing

This includes the first and last wake times and bedtimes, useful for assessing sleep schedule and alignment with personal circadian rhythms.

#### Sleep efficiency

The average ratio of total sleep time to total time spent in bed serves as an indicator of sleep quality.

#### Time in bed

The total and average time spent in bed, covering both sleep and awake periods, gives a measure of overall rest time.

### Step features

Step features are extracted to get insights into a student's level and consistency of physical activity. Maximum and minimum daily steps shed light on peak activity levels and periods of rest, respectively. The average daily steps provide a baseline of typical physical activity, while the median daily steps, being a robust measure of central tendency, can offer a more accurate depiction of daily activity especially in the presence of outliers. The standard deviation of daily steps quantifies the variability in physical activity, capturing fluctuations in day-to-day activity levels. Collectively, these features form a comprehensive representation of a student's physical activity patterns.

### Heart rate features

Heart rate features capture the important aspects of a student's cardiovascular health and fitness level. Resting heart rate metrics, including maximum, minimum, average, median, mode, and standard deviation, provide a comprehensive snapshot of heart function. The difference between the maximum and mode and between mode and minimum sheds light on the variability of the resting heart rate. The entropy measure offers a perspective on the diversity and unpredictability of resting heart rate values. Further, the calories burned within various heart rate zones highlight the intensity of physical exertion and its impact on caloric expenditure.

### Statistical analyses

#### Behavioural patterns and their relationship with loneliness and depression

First, we used the Shapiro–Wilk test to examine the normal distribution assumptions of the features.^25^ We used a logistic regression model to the StudentLife dataset to examine how lonely and depressed students’ behavioural features differ from their non-depressed and non-lonely counterparts. The logistic regression model was selected as the main statistical analysis technique because it is an effective method for investigating the relationship between a binary dependent variable (DV) and one or more independent variables (IVs). In this instance, the dependent variables were two measures of loneliness and depression (UCLA loneliness scale and PHQ-9 depression scale), while the independent variables were behavioural features retrieved from the StudentLife dataset using the RAPID framework.

With maximum likelihood estimation, the data was utilized to fit a logistic regression model. This gave us a method for determining the coefficients of the independent variables, which show how the log odds of the DV change when the independent variable goes up by one unit. The coefficients were used to generate the odds ratios, which show the likelihood of the DV given an increase of one unit in the IV. The odds ratios were interpreted to determine the degree and direction of the relationship between the independent and dependent variables. A p-value of <0.05 was used as an indicator of statistical significance.

In addition to the logistic regression model, a feature importance analysis was conducted to discover which behavioural characteristics were most essential in distinguishing between lonely and depressed students and other students. The analysis of feature relevance was conducted by rating the coefficients of the independent variables and choosing the highest-ranked features. This enabled us to identify the behavioural characteristics that were most strongly connected with loneliness and depression and hence the most crucial for understanding the prevalent behavioural features among lonely and depressed students. The findings of the logistic regression model and feature importance analysis will provide insight into the behavioural features that are more prevalent among students who are lonely and depressed compared to other students.

We used ARM to understand the combined behavioural patterns linked to loneliness and depression. This data mining technique identifies relationships and patterns within large datasets. In contrast to logistic regression, ARM evaluates the strength of associations among all possible combinations of behavioural patterns with loneliness and depression. It can reveal complex, multi-dimensional patterns that might not be evident with logistic regression alone.

We employed the Apriori algorithm, a popular ARM algorithm, to extract association rules from the dataset.^26^ It identifies frequent item sets—subsets of items appearing together often—by starting with the most frequent single items and gradually increasing the subset's size until all combinations are considered.

The Apriori algorithm's generated association rules were assessed using metrics like support, confidence, and lift. *Support* measures a behavioural pattern's occurrence frequency, *confidence* measures the association strength between the pattern and loneliness or depression, and *lift* compares the strength of associations between the pattern and loneliness/depression to the expected association if the features were independent. We set a minimum support threshold of 0.1, an 80% confidence level, and a lift value of 1.5 to filter out weaker associations and focus on the most relevant behavioural patterns.

To capture the nuances of different behavioural patterns, we divided each feature into three categories: low, medium, and high, using a binning technique. We calculated the 33rd and 66th percentiles of the data distribution, forming three bins for a balanced representation of each behavioural pattern. By converting features into categorical variables, we uncovered meaningful associations between combinations of these patterns and the outcomes of loneliness and depression in the ARM analysis.

Our goal was to explore the complex relationships between different behavioural features of loneliness and depression using ARM. By assessing the strength of these relationships, we can better understand the behavioural patterns common among lonely and depressed students, offering insights for further investigation.

#### Mediation analysis: understanding loneliness–depression interplay

We used mediation analysis to investigate the direct and indirect impacts of loneliness and depression on each other, as well as the particular behavioural features that serve as mediators in these relationships. Mediation analysis served as an important statistical technique for understanding the mechanism underlying the relationship between loneliness and depression. We were able to examine the direct and indirect effects of loneliness on depression and vice versa, as well as the behavioural features that may mediate these effects.^27^

In order to investigate the relationship between loneliness and depression, we conducted a mediation analysis using loneliness as the IV and depression as the dependent variable. Similarly, while studying the effect of depression on loneliness, depression served as the IV and loneliness as the DV. Each behavioural feature was investigated as a potential mediator variable. Before considering any mediator factors, we assessed the overall impact of the IV on the DV using a series of regression models. This stage allowed us to assess the general relationship between loneliness and depression, as well as the influence of IV on DV.

Next, we examined the indirect effects of the IV on the DV through mediator variables. To do this, regression analyses were undertaken to assess the effect of the IV on each possible mediator and the influence of each mediator on the DV. These analyses allowed us to identify the behavioural patterns that significantly mediated the connection between loneliness and depression. Finally, we assessed the direct effect of the IV on the DV by taking into account the indirect effects mediated by the mediator variables.

Our results for mediation analysis revealed a comprehensive understanding of the complicated relationship between loneliness and depression, as well as the significance of behavioural patterns in mediating this relationship. Calculating the total effect, direct effect, and indirect effect allowed us to evaluate the degree to which certain behavioural patterns mediated the relationship between loneliness and depression. In addition, the output included statistical significance values for each effect (total, direct, and indirect).

#### Machine learning prediction of loneliness and depression

In order to examine the effectiveness of digital biomarkers combined with loneliness or depression scales in predicting lonely and depressed groups, we utilized a variety of binary classification models, including logistic regression, k-nearest neighbours, support vector machine (SVM), random forest, gradient boosting, and extreme gradient boosting. These models were chosen due to their ability to handle both categorical and continuous features and their widespread use in similar research settings. We addressed the class imbalance in the training dataset using the synthetic minority oversampling technique (SMOTE).^28^ SMOTE generates synthetic data for the minority class, leading to a balanced training dataset. This method was applied to each student's data individually to tackle data imbalance on a per-client basis.

We compared the performance of the selected classification models using nested cross-validation. In the inner loop, threefold cross-validation was performed to tune hyperparameters, while in the outer loop, leave-one-subject-out cross-validation was used to evaluate the model's performance. This approach allowed us to evaluate the generalization ability of each model and minimize the risk of overfitting. To establish a baseline for comparison, we considered majority class, random weighted classifier, and decision tree models that used only the UCLA Loneliness Scale and PHQ-9 Depression Scale scores as input features. Performance metrics such as accuracy, precision, recall, F1-score, and area under the receiver operating characteristic curve (AUC) were calculated for each model and fold.

We trained each classification model on the preprocessed data, using the 82 extracted behavioural features and the loneliness and depression scores as input features. For each model, hyperparameters were tuned using grid search or random search techniques to optimize performance. This process ensured that we selected the best possible set of hyperparameters for each model, resulting in improved performance and a more reliable evaluation of the relationship between digital biomarkers, loneliness, and depression.

## Results

In this section, we present the significant findings of our study, which investigates the relationships between loneliness, depression, and behavioural features, as well as evaluates the performance of various machine learning models for predicting loneliness and depression.

### Identified behavioural features of lonely and depressed students

The results of the regression analysis reveal several significant associations between behavioural features and the loneliness and depression scores in Table 1. The following sections discuss the main findings for each behavioural feature.

**Table 1.** Results of regression analysis for loneliness and depression: odds ratios, 95% confidence intervals, and p-values for behavioural features.

	Loneliness			Depression		
Behavioural feature	Odds ratio	95% CI	p-value	Odds ratio	95% CI	p-value
Total number of screen unlocks	1.20	(1.05, 1.37)	0.031	0.75	(0.62, 0.91)	0.044
Number of screens unlocks (evening)	1.75	(1.32, 2.31)	0.001	0.80	(0.65, 0.95)	0.021
Phone usage duration	1.50	(1.20, 1.88)	0.003	0.70	(0.52, 0.94)	0.018
Phone usage duration (evening)	1.45	(1.15, 1.85)	0.002	1.10	(0.95, 1.26)	0.310
Number of places visited	1.73	(1.30, 2.20)	0.016	0.65	(0.50, 0.95)	0.034
Number of unique places visited	1.13	(1.03, 1.23)	0.021	0.85	(0.76, 0.95)	0.023
Activity duration	0.72	(0.54, 0.91)	0.014	0.69	(0.55, 0.87)	0.022
Sleep duration	0.46	(0.30, 0.70)	0.002	0.75	(0.62, 0.90)	0.003
Number of conversations	1.50	(0.95, 2.38)	0.101	0.75	(0.63, 0.89)	0.002
Number of incoming calls	0.82	(0.71, 0.94)	0.023	1.10	(0.90, 1.35)	0.351
Number of incoming calls (evening)	0.30	(0.20, 0.45)	0.002	1.10	(0.95, 1.26)	0.303
Unique Bluetooth scans during day	1.20	(0.95, 1.50)	0.204	0.50	(0.35, 0.70)	0.003

#### Phone usage

This section refers to general smartphone usage, e.g., what type of apps a user is using such as social apps, games, and productivity apps. In our study, we found a strong relationship between phone usage duration and loneliness. The odds ratio was 1.50 (95% confidence interval (CI): 1.20–1.88, p = 0.003), indicating that increased phone usage is associated with higher chances of experiencing loneliness.

There are different findings for the relationship between overall phone usage duration and depression. The odds ratio was 0.70 (95% CI: 0.52–0.94, p = 0.018), implying that higher phone usage duration is associated with a decreased likelihood of depression.

When we looked at the phone usage duration in the evening, the link to loneliness was still there, with an odds ratio of 1.45 (95% CI: 1.15–1.85, p = 0.002). This finding backs up the idea that using the phone more in the evening is also linked to a higher chance of feeling lonely. But the link between phone usage duration and depression was not statistically significant.

#### Locations

We found that the overall number of places visited throughout a day was positively associated with loneliness, with an odds ratio of 1.73 (95% CI (1.30, 2.20), p = 0.016). However, when considering the number of unique places visited during a day, the association with loneliness remained positive but weaker, with an odds ratio of 1.13 (95% CI (1.03, 1.23), p = 0.021).

In contrast, we observed a negative association between the overall number of places visited throughout a day and depression, with an odds ratio of 0.65 (95% CI (0.50, 0.95), p = 0.034). However, when examining the number of unique places visited during a day, the association with depression was negative but not statistically significant, with an odds ratio of 0.85 (95% CI (0.76, 0.95), p = 0.023).

#### Physical activity

For loneliness, we observed an odds ratio of 0.72 (95% CI: 0.54–0.91, p = 0.014). Similarly, for depression, the analysis revealed an odds ratio of 0.69 (95% CI: 0.55–0.87, p = 0.022).

#### Sleep

For loneliness, the odds ratio was found to be 0.46 (95% CI: 0.30–0.70, p = 0.002), which means that a longer duration of sleep is linked to a 54% lower chance of being lonely. In the case of depression, the odds ratio was found to be 0.75 (95% CI: 0.62–0.90, p = 0.003).

#### Conversation

Our regression analysis revealed a significant relationship between conversation duration and depression, yielding an odds ratio of 0.75 (95% CI: 0.63–0.89, p = 0.002). However, the relationship between conversation duration and loneliness was not found to be statistically significant.

#### Calls/SMS

Our regression analysis revealed a significant negative association between the number of incoming calls throughout the day and loneliness, with an odds ratio of 0.82 (95% CI: 0.71–0.94, p = 0.023). Additionally, the number of incoming calls in the evening showed a pronounced odds ratio of 0.30 (95% CI: 0.20–0.45, p = 0.002) for loneliness. Conversely, the relationships between the number of calls and depression, both during the day and in the evening, were not found to be statistically significant.

#### Bluetooth

Bluetooth scans were used in our study as a proxy for detecting the presence of other people, since Bluetooth technology allows devices to detect nearby Bluetooth-enabled devices. In our regression analysis, we found that the number of unique Bluetooth scans throughout a day was not statistically significant in predicting loneliness, with an odds ratio of 1.20 (95% CI (0.95, 1.50), p = 0.204). However, when considering the association with depression, we observed a strong negative association, with an odds ratio of 0.50 (95% CI (0.35, 0.70), p = 0.003), suggesting that an increase in the number of unique Bluetooth scans throughout a day is associated with a 50% decrease in the odds of experiencing depression. This finding highlights the potential protective role of increased social interactions, as inferred from the unique Bluetooth scans, against the development of depression.

#### WiFi

No significant associations were found between any WiFi features and loneliness or depression scores.

Our regression analysis identified several behavioural patterns that are significantly associated with loneliness and/or depression. Increased phone usage, evening phone usage, and overall number of places visited were associated with higher levels of loneliness, while higher physical activity, sleep duration, and the number of incoming calls were associated with lower levels of loneliness. Depression was found to be negatively associated with phone usage duration, overall number of places visited, physical activity, sleep duration, conversation duration, and the number of unique Bluetooth scans, while no significant association was observed for WiFi features. It is important to note that some patterns, such as overall phone usage duration and the number of incoming calls, showed contrasting effects on loneliness and depression.

The ARM analysis revealed several interesting patterns of behaviours associated with loneliness among students in Tables 2 and 3. The most notable rule (Rule 1) showed that students who had a high number of screen unlocks during the evening, high overall phone usage duration, and high phone usage duration during the evening were more likely to experience loneliness. This finding is consistent with the regression analysis results, which also showed significant positive associations between loneliness and these behavioural features. Another important rule (Rule 2) identified a combination of low sleep duration, low number of incoming calls during the evening, and a high number of unique places visited as being related to loneliness. The ARM results for depression revealed fewer distinct behavioural patterns compared with those found for loneliness. Rule 1 for depression showed that students who had a low total number of screen unlocks, low phone usage duration, and low number of places visited were more likely to experience depression. This finding is in line with the regression analysis results, which also indicated significant negative associations between depression and these behavioural features. Another important rule (Rule 2) found a combination of low activity duration, low number of incoming calls, and low unique Bluetooth scans during the day as being related to depression. This suggests that students who have less physical activity, fewer social interactions, and less exposure to diverse social environments may be at a higher risk of depression.

**Table 2.** Results of association rule mining for loneliness: support, confidence, lift, and behavioural patterns.

Rule behavioural pattern	Support	Confidence	Lift
1. High evening phone usage, high evening screen unlocks, low conversations, low activity duration, low sleep, low evening incoming calls	0.12	0.85	1.65
2. High total screen unlocks, high unique places visited, low sleep, low incoming calls, low activity duration, medium conversations	0.11	0.82	1.59
3. High phone usage, medium total screen unlocks, high evening screen unlocks, low sleep, low conversations, low day Bluetooth scans	0.13	0.88	1.71
4. Low places visited, low activity duration, high total screen unlocks, low sleep, medium incoming calls, low day Bluetooth scans	0.14	0.84	1.63
5. Low places visited, low unique places visited, high evening phone usage, low sleep, low conversations, high evening screen unlocks	0.10	0.81	1.57

**Table 3.** Results of association rule mining for depression: support, confidence, lift, and behavioural patterns.

Rule behavioural pattern	Support	Confidence	Lift
1. High phone usage, high screen unlocks, low places visited, low unique places visited, low activity duration, low day Bluetooth scans	0.11	0.83	1.62
2. Low sleep, high total screen unlocks, low incoming calls, low conversations, low activity duration, high evening screen unlocks	0.12	0.86	1.67
3. Low places visited, low unique places visited, high phone usage, high evening screen unlocks, low activity duration, medium incoming calls	0.14	0.81	1.58
4. Low sleep, low conversations, high total screen unlocks, low incoming calls, low activity duration, high evening screen unlocks	0.10	0.80	1.55

### How loneliness and depression affect each other

The mediation analysis results for loneliness and depression are presented in Table 4. Overall, the findings provide evidence for both direct and indirect effects on the relationship between loneliness and depression and vice versa. The total effect of loneliness on depression was found to be statistically significant (estimate = 1.48, p = 0.001), suggesting that loneliness increases the likelihood of depression by 48%. The direct effect of loneliness on depression was also significant (estimate = 1.25, p = 0.041), indicating that loneliness directly increases the likelihood of depression by 25% when considering the influence of various mediators. In contrast, the total effect of depression on loneliness was not statistically significant (estimate = 1.10, p = 0.230), suggesting that depression might not directly lead to a substantial increase in loneliness. The direct effect of depression on loneliness was also not significant (estimate = 1.05, p = 0.480).

**Table 4.** Mediation analysis results for loneliness and depression.

Effect	Loneliness → depression			Depression → loneliness		
	Estimate	95% CI	p-value	Estimate	95% CI	p-value
Total effect	1.48	(1.22, 1.80)	0.001	1.10	(0.85, 1.50)	0.230
Direct effect	1.25	(0.98, 1.59)	0.041	1.05	(0.80, 1.40)	0.480
Indirect effect (number of screen unlocks during day)	1.10	(1.02, 1.18)	0.055	0.80	(0.75, 0.85)	0.262
Indirect effect (number of screen unlocks during evening)	1.75	(1.32, 2.31)	0.001	0.80	(0.65, 0.95)	0.021
Indirect effect (phone usage duration)	1.50	(1.20, 1.88)	0.003	0.70	(0.52, 0.94)	0.018
Indirect effect (phone usage duration during evening)	1.45	(1.15, 1.85)	0.002	1.10	(0.95, 1.26)	0.310
Indirect effect (number of places visited)	1.73	(1.30, 2.20)	0.116	0.65	(0.50, 0.95)	0.034
Indirect effect (number of unique places visited)	1.51	(1.15, 1.87)	0.038	0.74	(0.45, 0.80)	0.085
Indirect effect (activity duration)	0.72	(0.54, 0.91)	0.014	0.69	(0.55, 0.87)	0.022
Indirect effect (sleep duration)	0.46	(0.30, 0.70)	0.002	0.75	(0.62, 0.90)	0.003
Indirect effect (number of conversations)	1.07	(0.98, 1.16)	0.044	0.99	(0.94, 1.04)	0.250
Indirect effect (number of conversations during day)	1.40	(0.85, 2.25)	0.121	0.76	(0.62, 0.92)	0.004
Indirect effect (total number of incoming calls)	0.98	(0.92, 1.04)	0.276	0.98	(0.95, 1.01)	0.165
Indirect effect (total number of unique Bluetooth scans)	1.02	(0.96, 1.08)	0.390	0.96	(0.91, 1.01)	0.110

For loneliness leading to depression, significant indirect effects were observed for various behavioural features. A one-unit increase in evening screen unlocks was associated with a 1.75 times increase in depression, considering the role of loneliness, and this effect was statistically significant. Similarly, a one-unit increase in phone usage duration was linked to a 1.50 times increase in depression when accounting for loneliness, and this effect was also statistically significant. When examining phone usage duration during the evening, a one-unit increase was found to relate to a 1.45 times increase in depression when considering the effect of loneliness, and this effect was statistically significant as well. For the number of unique places visited, a one-unit increase was connected to a 1.73 times increase in depression after taking into account the influence of loneliness, and this effect was statistically significant. In contrast, increased activity duration and sleep duration showed protective effects against depression. A one-unit increase in activity duration was associated with a 0.72 times decrease in depression when accounting for loneliness, and this effect was statistically significant. Likewise, a one-unit increase in sleep duration was linked to a 0.46 times decrease in depression when considering the role of loneliness, and this effect was statistically significant, suggesting that longer sleep duration is protective against depression.

Significant indirect effects were observed for various behavioural features in the context of depression leading to loneliness. A one-unit increase in evening screen unlocks was associated with a 0.80 times decrease in loneliness when accounting for the effect of depression, and this effect was statistically significant. Likewise, a one-unit increase in phone usage duration was linked to a 0.70 times decrease in loneliness when considering the role of depression, and this effect was statistically significant as well. For the number of places visited and the number of unique places visited, a one-unit increase in either of these features was connected to a 0.65 times decrease in loneliness after taking into account the influence of depression, and both of these effects were statistically significant. Additionally, a one-unit increase in activity duration was associated with a 0.69 times decrease in loneliness when accounting for depression, and this effect was statistically significant. A one-unit increase in sleep duration was linked to a 0.75 times decrease in loneliness when considering the role of depression, and this effect was statistically significant, suggesting that longer sleep duration is protective against loneliness. Lastly, a one-unit increase in the number of conversations during the day was related to a 0.76 times decrease in loneliness after accounting for the effect of depression, and this effect was statistically significant as well.

### Machine learning predictive power for loneliness and depression

In this section, we present the results of our experiments on predicting loneliness and depression using various machine learning models. The performance of these models is evaluated based on several metrics, including accuracy, area under the curve (AUC), FI macro, and F1 scores for both classes (1 and 0).

Table 5 shows the performance of different classification models for predicting loneliness. The XGBoost model achieved the highest accuracy of 82.43%, with an AUC of 83.31% and an FI macro of 74.34%. In comparison, the SVM model came in second, with an accuracy of 76.90%, an AUC of 75.89%, and an FI macro of 68.67%. The logistic regression model ranked third, with an accuracy of 66.84%, an AUC of 70.26%, and an FI macro of 63.30%. The K-nearest neighbours, random forest, and three baseline models showed relatively lower performance across all metrics.

**Table 5.** Performance of classification models for loneliness prediction. The model performances are compared with three baseline classifiers (MC, majority class; RWC, random weighted classifier; and DT, decision tree). All values are reported as percentages.

Models	Accuracy	AUC	FI macro	Precision1	Recall1	F11	Precision0	Recall0	F10
Baseline 1: MC	53.05	50.00	42.55	0.00	0.00	0.00	63.78	100.00	86.39
Baseline 2: DT	51.53	47.48	48.38	26.29	20.38	23.26	65.58	82.18	71.39
Baseline 3: RWC	58.72	52.64	50.88	32.48	24.21	28.64	71.39	76.39	74.82
Logistic regression	66.84	70.26	63.30	49.73	58.82	54.92	80.18	73.19	77.33
Support vector machine	76.90	75.89	68.67	58.25	54.77	56.36	84.54	86.48	85.48
K-nearest neighbours	68.25	69.35	64.12	42.93	65.76	51.95	85.12	69.13	76.30
Random forest	70.82	68.08	62.11	44.06	43.84	43.95	80.21	80.35	80.28
XGBoost	82.43	83.31	74.34	70.97	60.49	65.36	84.17	91.28	87.18

Table 6 presents the performance of the classification models for depression prediction. Similar to the loneliness prediction results, the XGBoost model outperformed all other models, achieving an accuracy of 79.43%, an AUC of 80.21%, and an FI macro of 71.24%. The SVM model ranked second, with an accuracy of 74.60%, an AUC of 73.49%, and an FI macro of 65.57%. The logistic regression model came in third, with an accuracy of 67.81%, an AUC of 66.21%, and an FI macro of 59.20%. The K-nearest neighbours and random Forest models demonstrated lower performance across all metrics. The baseline models, as expected, performed the worst among all models under consideration. These results are consistent with our earlier work on loneliness detection.^32^

**Table 6.** Performance of classification models for depression prediction. The model performances are compared with three baseline classifiers (MC, majority class; RWC, random weighted classifier; and DT, decision tree). All values are reported as percentages.

Models	Accuracy	AUC	FI macro	Precision1	Recall1	F11	Precision0	Recall0	F10
Baseline 1: MC	69.07	50.00	40.55	0.00	0.00	0.00	69.07	100.00	81.61
Baseline 2: DT	56.26	45.96	43.96	18.43	19.43	18.93	69.50	68.50	69.00
Baseline 3: RWC	58.68	48.13	47.88	23.07	24.14	23.75	70.15	71.12	70.61
Logistic regression	67.81	66.21	59.20	45.61	52.11	48.68	79.26	74.86	76.93
Support vector machine	74.60	73.49	65.57	53.15	47.67	50.26	81.54	83.38	82.43
K-nearest neighbours	65.15	66.25	60.02	39.83	58.66	47.85	82.02	67.03	73.90
Random forest	67.72	65.98	58.01	41.96	40.74	41.35	77.11	78.25	77.68
XGBoost	79.43	80.21	71.24	65.87	56.39	60.86	83.99	89.25	86.52

## Discussion

Our study investigated the associations between behavioural features and loneliness and depression among students, revealing significant insights that have critical implications for developing targeted interventions and early detection methods. Understanding these patterns can enable timely intervention and support, potentially improving people's mental health.

### Interrelatedness of loneliness and depression

While loneliness and depression are distinct mental health conditions, they often co-occur and can influence each other. The interrelatedness of these two conditions is evident in our findings, as some behavioural features were found to be associated with both loneliness and depression. Increased physical activity duration throughout the day was significantly associated with reduced odds of both loneliness and depression. This suggests that engaging in physical activities may have a protective effect against both, emphasizing the importance of promoting regular activities for overall mental health and well-being. Similarly, increased sleep duration was found to be significantly associated with reduced odds of experiencing both loneliness and depression. It also revealed that longer sleep duration is linked to a 25% lower chance of having depression. These results show that getting more sleep is strongly linked to a lower chance of both being lonely and being depressed. Our findings are consistent with the existing research.^30,31^ This highlights the importance of healthy sleep habits in protecting against mental health issues and underscores the need for interventions targeting sleep hygiene to address loneliness and depression among students.

Despite the interrelatedness of loneliness and depression, our findings revealed distinct behavioural features associated with each condition. Phone usage duration was found to have a complex relationship with both loneliness and depression. Increased phone usage duration throughout the day was associated with an increased likelihood of loneliness, but a decreased likelihood of depression. These patterns could potentially be explained by the different ways individuals use their phones to cope with loneliness or depression. It is possible that lonely individuals might use their phones to pass their time, whereas those with depression may find relief or support through phone-based activities or connections. However, further research is needed to better understand the underlying mechanisms and validate this interpretation. Additionally, the number of incoming calls throughout the day was negatively associated with loneliness, but no significant association was found with depression. This suggests that receiving more incoming calls might help reduce feelings of loneliness; however, this association does not appear to have a direct impact on depression. Further investigation is required to better understand the nuances of these relationships and to determine the specific effects of incoming calls on loneliness and depression. The number of unique Bluetooth scans throughout a day was not significantly associated with loneliness, but it showed a strong negative association with depression, highlighting the potential protective role of increased social interactions inferred from Bluetooth scans. ARM revealed several interconnected behavioural patterns that were associated with loneliness and depression. These patterns included high phone usage duration, low sleep duration, and low physical activity, among others. These findings are in line with the existing research.^29–31^ Our findings also demonstrated the importance of social interactions, as evidenced by the relationship between the number of incoming calls and emotional well-being.

Another observation suggests that a greater overall number of places visited throughout a day may be associated with increased loneliness, while the number of unique places visited during a day may be a more nuanced predictor of loneliness. On the other hand, a greater overall number of places visited throughout a day may be associated with decreased depression, while the number of unique places visited during a day may not be a significant predictor of depression. This observation might seem paradoxical at first instance, as one could assume that increased social interaction and outdoor activity, inferred from the number of places visited, would correlate with decreased feelings of loneliness. However, similar findings have been reported in previous literature. For instance, a study found the same relationship, which supports our observation.^33^ It's worth considering that the act of visiting many different places might be indicative of a search for social connection that the individual isn’t finding in their usual environments, thus contributing to feelings of loneliness. Furthermore, the act of going to many places could also reflect an individual's coping mechanism to combat feelings of loneliness, trying to stay occupied or busy to avoid acknowledging these feelings. As a result, individuals experiencing loneliness may end up visiting more places but may not necessarily have meaningful or satisfying social interactions at these places.

### Novel insights and existing literature

These complex and nuanced findings address our research question by highlighting the interconnected nature of students’ behaviours and their mental health. By exploring these associations, we can better understand the factors contributing to loneliness and depression in students and identify potential areas for intervention. This provided a more comprehensive understanding of the relationships between behavioural patterns and mental well-being. While the regression analysis identified individual behavioural features that were significantly associated with loneliness and depression, ARM uncovered the complex interplay between multiple behaviours, demonstrating the importance of considering their combined effects. This multi-method approach allowed us to gain deeper insights into the specific patterns of behaviour that are more prevalent in lonely and depressed students compared to their peers.

The mediation analysis played a vital role in deepening our understanding of the relationship between loneliness and depression by investigating the indirect effects of various behavioural factors. This approach allowed us to explore the complex interplay between these mental health conditions and associated behaviours, thereby providing a more comprehensive picture of their interconnectedness. By identifying significant indirect effects in the relationship between loneliness and depression and vice versa, we were able to gain insights into how specific behaviours can mediate this relationship. For example, the mediation analysis revealed that evening screen unlocks, phone usage duration, and the number of unique places visited were significant mediators in the relationship between loneliness and depression, aligning with insights from traditional studies. What distinguishes our study is the methodology; we affirmed these findings utilizing passive sensing data, a departure from conventional approaches such as questionnaires. This suggests that these behaviours may play a crucial role in the transition from loneliness to depression or vice versa and could be potential targets for interventions. Similarly, the mediation analysis highlighted the importance of activity duration and sleep duration as protective factors against both loneliness and depression. Furthermore, the mediation analysis extended our understanding of the role of social interactions in the relationship between loneliness and depression. For instance, the number of conversations during the day was found to be a significant mediator in the relationship between depression and loneliness. This finding underscores the importance of fostering social connections as a means of reducing the risk of both loneliness and depression. To the best of our knowledge, this is the first time that mediation analysis has been employed to investigate the role of behavioural factors in the relationship between loneliness and depression using passive sensing data. Our findings can serve as a foundation for future research in this area, potentially leading to more effective interventions and support strategies for students facing mental health challenges.

The findings are also consistent with existing research that emphasizes the importance of monitoring and managing screen usage, sleep, physical activity, and social interactions to maintain psychological well-being. High screen usage, particularly in the evenings, has been linked to feelings of depressive symptoms.^34^ This finding also aligns with the positive relationship between phone usage duration and loneliness (OR = 1.50, 95% CI (1.20, 1.88), p = 0.003), which has been demonstrated in prior research.^29^ Additionally, low sleep, low activity duration and low social interaction have been found to exacerbate loneliness and depression.^30,31,35,36^ Engaging in diverse activities and visiting various locations may help reduce feelings of loneliness, although the complexity of this relationship may be influenced by individual preferences and social context. Our study adds to existing research that emphasizes the importance of monitoring and managing screen usage, sleep, physical activity, and social interactions to maintain psychological well-being.

Our study stands out by providing more granular behavioural information using a dataset that was collected through passive, real-time mobile sensing, overcoming the limitations of self-report methods typically used in previous studies. The real-time nature of the sensing helps to detect changes in loneliness and depression continuously, allowing for more timely application of interventions or evaluation of their efficacy in a personalized manner, which is in line with the concept of personalized health care. Our findings, such as the positive relationship between phone usage duration and loneliness, align with prior research but offer more detailed insights into these associations. Additionally, our study highlights the complexities in the relationship between engaging in diverse activities, visiting various locations, and feelings of loneliness, which may be influenced by individual preferences and social context. In addition, we have shown the importance of using multiple methods to validate our results, showing the potential for innovative research that goes beyond traditional disciplinary boundaries. By combining perspectives and methodologies from multiple disciplines such as digital health, psychology, and data science, we have not only reinforced our findings but also highlighted the importance of interdisciplinary collaboration to better understand complex human experiences. This approach emphasizes the growing role of digital health tools in enabling and enhancing personalized care and support for individuals.

## Limitations and future work

In acknowledging the limitations of this study, we must first recognize that our findings require validation on a larger and, notably, more diverse population sample. The participants in our study were college students who may share similar daily routines and psychological pressures, limiting the spectrum of potential influential factors in our analysis. As such, the results’ generalizability may be confined within these circumstances. Furthermore, while our study offers unique insights into the interplay of loneliness and depression among college students, we acknowledge that the dataset used may not fully capture all pertinent factors affecting these conditions. Notably, aspects such as personal relationships, family history, significant life events, mental health history, or individual coping mechanisms, which could considerably shape an individual's experience of loneliness and depression, are not included in the StudentLife dataset. It is important to note that the results of our work are limited by this specific dataset, as it includes data from only 41 students. It also means that our findings apply only to this particular group of participants and may not be generalizable to everyone. Therefore, while our findings illuminate the associations between sensor data and feelings of loneliness and depression, they should be viewed in the context of these limitations. Future research could consider incorporating these additional factors to achieve a more comprehensive understanding of these conditions among college students.

## Conclusion

This study has provided insights into the nuanced associations between college students’ behavioural patterns and their experiences of loneliness and depression. We found that both increased physical activity and longer sleep duration serve as protective factors against these conditions. It is notable that while loneliness and depression have some common behavioural tendencies, each manifests unique behavioural patterns as well. Specifically, lonely students tend to increase their phone usage in the evening, perhaps seeking connection or distraction, while those experiencing depression show reduced screen interactions and limited mobility. The mediation analysis underscored that loneliness can elevate the risk of depression by a significant 48%. The analysis further shows the complex relationships between loneliness, depression, and behavioural patterns. For instance, increases in evening screen unlocks and phone usage duration significantly increase the risk of depression, while longer physical activity duration and more day-time conversations notably reduced feelings of loneliness and depression. Our machine learning models, especially the XGBoost model, effectively showcased the potential of passive sensing data in predicting mental health states among students. Our findings reveal important insights into mental health, highlighting the effects of screen use, sleep, and social interactions. These findings emphasize the need for targeted mental health solutions based on specific behaviours linked to loneliness and depression. By combining digital health, psychology, and machine learning, we offer deeper insights and a foundation for improved interventions to boost student well-being.
